# Evaluation of Patient-Initiated Follow-Up (PIFU) Service in a Fracture Clinic: A Comprehensive Service Evaluation and Patient Satisfaction Audit

**DOI:** 10.7759/cureus.73461

**Published:** 2024-11-11

**Authors:** Zubair Younis, Muhammad A Hamid, Muhammad Murtaza Khan, Rahul Sapra, Gurukiran Gurukiran, Rohit Singh

**Affiliations:** 1 Orthopaedics, Royal Shrewsbury Hospital, Shrewsbury, GBR; 2 Orthopaedic Surgery, University Hospitals Birmingham, Birmingham, GBR; 3 Trauma and Orthopaedics, Princess Royal Hospital Telford, Telford, GBR; 4 Trauma and Orthopaedics, Walsall Manor Hospital, Walsall, GBR; 5 Orthopaedics and Trauma, Royal Shrewsbury Hospital, Shrewsbury, GBR; 6 Orthopaedics, Shrewsbury and Telford Hospitals NHS Trust, Shrewsbury, GBR

**Keywords:** fracture clinic follow-up, healthcare resource management, outpatient care optimization, patient-initiated follow-up (pifu), patient satisfaction audit

## Abstract

Background

Outpatient clinics are increasingly challenged by high patient volumes and rising "did not attend" (DNA) rates, leading to extended wait times and declines in patient satisfaction. Traditional follow-up (FU) models with routinely scheduled appointments contribute to inefficiencies, as stable patients may attend unnecessary visits, thus straining clinic resources. The patient-initiated follow-up (PIFU) model offers an alternative where patients schedule appointments only when necessary. This study evaluates PIFU's efficacy in improving outpatient services and patient satisfaction by reducing routine appointments and prioritizing patient-driven follow-up.

Methods

This service evaluation and patient satisfaction audit was conducted at the fracture clinic of Royal Shrewsbury Hospital over three months (December 2023-March 2024). Out of 3828 patients seen, 203 were assigned to PIFU based on criteria indicating stable conditions with minimal follow-up requirements. The remaining patients were either scheduled for routine follow-ups or discharged. Data were collected retrospectively from clinic records, and a structured questionnaire assessed patient satisfaction with the PIFU service.

Results

Among the 203 patients assigned to PIFU, 183 (90.15%) patients received an informational leaflet, with all respondents finding it easy to understand. However, only 41 (20.2%) of patients utilized the PIFU service, primarily for concerns about pain, healing, or complications. Satisfaction among PIFU users was high, with 163 (80.3%) patients rating the service 5/5. Non-users mostly cited no perceived need for follow-up. Demographic analysis indicated that patients aged 40-60 were predominant (n=132; 65.02%) among the PIFU cohort.

Conclusion

The PIFU model demonstrated the potential to alleviate clinic workload by reducing routine follow-ups while maintaining high patient satisfaction. Although utilization rates were low, those who engaged found the service beneficial, suggesting PIFU's value for patients comfortable with self-management. Improved patient education may enhance engagement, supporting the broader implementation of PIFU in outpatient settings. Further research is warranted to explore barriers to patient-initiated follow-up and refine eligibility criteria for optimal outcomes.

## Introduction

Outpatient services face increasing pressure to manage rising patient volumes while maintaining high standards of care. The number of annual outpatient appointments, the majority of which are for routine follow-up care, has surged to 119.4 million per year, doubling in just a decade [1]. This surge, reflecting global patterns, has placed substantial strain on hospital finances and staff resources [2]. It has also been accompanied by a marked increase in missed appointments, or 'did not attend' (DNAs). The DNA rate has risen by 44% in under a decade, prompting many departments to overbook patients. This approach is further worsening long wait times and contributing to declining patient satisfaction [1].

Current models are often regarded as inflexible in addressing patient needs, prompting calls for alternative follow-up approaches that are more tailored, adaptable, and cost-effective [3]. Traditional follow-up (FU) models, which rely on routinely scheduled appointments, often lead to inefficiencies as patients with stable conditions may attend unnecessary visits, straining resources and contributing to longer waiting times and clinic backlogs. These challenges have prompted the exploration of alternative approaches to improve both patient care and resource management.

In response to these challenges, the patient-initiated follow-up (PIFU) model has emerged as a promising alternative, which has been adopted by various NHS trusts to address these issues. A PIFU model offers an alternative approach, where patients schedule follow-up appointments based on their specific needs and symptoms ('on-demand'), rather than adhering to a fixed appointment schedule [4]. This model allows for rapid access to specialist care when necessary, while routine clinic appointments are either reduced or no longer regularly scheduled, particularly in specialities like trauma and orthopaedics, where long-term management of conditions such as fractures and joint replacements is common. Decreasing the number of scheduled follow-up appointments may improve clinic capacity, allowing for greater accessibility to 'on-demand' appointments and more responsive patient care [5]. By reducing unnecessary clinic reviews, PIFU can improve resource allocation and clinic efficiency, while also potentially reducing patient anxiety associated with routine follow-up visits [6]. Patients are given guidance on recognizing signs and symptoms of recurrence and are instructed to contact a designated healthcare professional if they experience specific symptoms, as routine appointments are not scheduled under the PIFU model [4,7].

Despite its promise, there remains some uncertainty about the overall effectiveness and cost-efficiency of PIFU [4]. It is believed that PIFU may reduce diagnostic delays by providing patients with more rapid access to specialist care when needed [5]. However, the model also carries risks, as it relies on patients’ ability and willingness to self-manage their symptoms and initiate contact when necessary [8]. This could lead to missed symptoms or delayed interventions if patients do not seek care promptly.

In trauma and orthopaedic services, the potential benefits of the PIFU model must be carefully weighed against its risks. While PIFU may alleviate clinic overcrowding and increase capacity for urgent cases, it is crucial that patients receive adequate guidance to recognize and respond to warning signs. Some patients may appreciate the flexibility and autonomy PIFU offers, while others may prefer the reassurance of regular, face-to-face follow-ups with their healthcare provider.

This study assesses the implementation of the PIFU model at the fracture clinic of the Royal Shrewsbury Hospital, a key part of a larger initiative to optimize outpatient care.

## Materials and methods

This study was a service evaluation and patient satisfaction audit conducted in the fracture clinic of Royal Shrewsbury Hospital (RSH), with SMART objectives (Specific, Measurable, Achievable, Relevant, and Time-Bound) guiding its design. The primary objective was to achieve a satisfaction rate of at least 80% among patients assigned to the PIFU service. The aim was to assess both the operational outcomes of the PIFU system and patient satisfaction with this service over a time-bound three-month period, from December 2023 to March 2024.

During this period, the clinic aimed to optimize follow-up practices by reducing unnecessary appointments and providing a more patient-centred approach through the PIFU system. Out of the 3,828 patients who attended the fracture clinic during the study period, 203 patients, selected based on clinical criteria emphasizing stability and low risk of complications, were assigned to the PIFU service. This selection focused on patients who would only need further follow-up if they experienced specific complications or concerns.

Specific inclusion and exclusion criteria

Patients were included in the PIFU service based on clinical judgments about the stability of their fracture or musculoskeletal condition, aligning closely with clinic objectives for optimizing resources and enhancing patient autonomy. Patients with complex fractures requiring frequent check-ups or imaging were excluded to keep the study’s focus on cases more amenable to patient-initiated follow-ups.

To provide clarity, Table 1 below outlines the specific conditions and associated follow-up criteria used to determine eligibility for the PIFU program:

**Table 1:** Conditions Eligible for Patient-Initiated Follow-Up (PIFU) Based on Visit Type Note: Hand injuries, such as volar plate injuries and phalangeal fractures, were additionally followed up with hand therapists. This extra step supported optimal recovery and maximized hand function through specialized rehabilitation for these cases.

Patient Follow-Up Visit	Conditions Included in Patient-Initiated Follow-Up (PIFU)
1st Visit	Undisplaced wrist fractures, undisplaced radial head fractures, low ankle sprains, acute osteoarthritis flare, volar plate hand injuries, acromioclavicular (AC) joint injuries (Rockwood Type I, II and III), lateral clavicle fractures (Neer's type I and IIa), distal biceps tendon injuries (conservatively managed), isolated stable fibular shaft fractures, phalangeal fractures (finger fractures without rotation deformity)
2nd Visit	5th metatarsal base fractures (undisplaced), proximal humerus fractures (conservatively managed), medial collateral ligament (MCL) knee injuries (Grade 1 and 2), stable lateral malleolar fractures, shaft of clavicle fractures (conservatively managed)

Data collection and analysis

Data were retrospectively collected from clinic records for all 3,828 patients, documenting discharge status, scheduled follow-ups, or assignment to the PIFU system. For the 203 patients in PIFU, a structured satisfaction questionnaire was administered during routine phone calls or clinic visits. This questionnaire included questions about the clarity of instructions and overall experience with the PIFU model, as detailed below (Table 2).

**Table 2:** Patient Satisfaction Questionnaire

S. No.	Questionnaire	
1,	Did you receive a leaflet explaining the PIFU service?	Yes/No
2.	Was the leaflet easy to understand?	Yes/No
3.	Have you used the PIFU service?	Yes/No
4.	Were you provided with satisfactory advice over the telephone or given an appropriate appointment?	Yes/No
5.	On a scale of 1-5, how would you rate the PIFU service?	1-5

Data collection and analysis

In addition to satisfaction data, demographic (age, gender) and clinical data (type of fracture or condition) were collected. This enabled analysis of trends in PIFU engagement across different demographics. Descriptive statistics summarized the outcomes: percentages of patients discharged, scheduled for follow-ups, or assigned to PIFU were calculated and compared. Patient satisfaction was evaluated based on the percentage who received the leaflet, found it easy to understand, and rated their PIFU experience on a 1-5 scale.

As this was a service evaluation designed to improve clinical practices and patient care, formal ethical approval was not required. However, all patients involved were informed of the evaluation, and their participation in the satisfaction questionnaire was voluntary. Data were anonymized, and patient confidentiality was maintained in line with hospital guidelines.

## Results

Out of the 3828 patients who attended the fracture clinic during the study period, 203 patients were selected to be part of the PIFU service. The remaining patients were either discharged from the clinic after their treatment or scheduled for follow-up appointments (Table 3). 

**Table 3:** Patient Allocation and Follow-Up Status in Fracture Clinic PIFU = Patient Initiated Follow-Up

Category	Number	Percentage (%)
Patients allotted to PIFU	203	5.3
Patients allotted regular follow-up	2549	66.6
Patients discharged	1076	28.1
Total patients attending fracture clinic	3828	100

Among the 203 patients assigned to the PIFU service, only 183 (90.15%) received the leaflet (Table 4). The demographics of the patients were diverse, ranging in age from 18 to 75 years, with the majority of patients 132 (65.02%) aged between 40 and 60. The gender distribution was relatively balanced, with 118 male (58.12%) and 85 female (41.87%) patients (Table 5). The conditions qualifying for PIFU pathways primarily included stable fractures, soft tissue injuries, and routine postoperative follow-ups, such as those following elective joint procedures or minor surgical interventions. The majority of patients had stable fractures that did not require routine clinical imaging or physical follow-ups, making them ideal candidates for self-initiated follow-ups. 

**Table 4:** Distribution of PIFU Service Leaflets PIFU: patient-initiated follow-up

Category	Number	Percentage (%)
Patients who received a leaflet	183	90.15
Patients who did not receive leaflets	20	9.85
Total patients assigned to PIFU	203	100

**Table 5:** Demographics of Patients Assigned to PIFU PIFU: patient-initiated follow-up

Demographic Category	Details	Number	Percentage (%)
Age range	18 - 75 years	203	-
Predominant age group	40 - 60 years	132	65.02
Gender distribution	Male	118	58.12
	Female	85	41.87

Utilization of PIFU

Despite the cohort of 203 patients assigned to the PIFU service, only 41 (20.2%) patients actively used the service to initiate follow-up appointments. These follow-ups were primarily requested due to ongoing pain, concerns about healing, or perceived complications such as swelling, stiffness or reduced mobility. The remaining 162 (79.8%) patients did not initiate any follow-ups, suggesting that their conditions were stable and did not necessitate further clinical intervention (Table 6).

**Table 6:** Patient Engagement with PIFU Service PIFU: patient-initiated follow-up

Utilization Status	Number of Patients	Percentage (%)
Actively used PIFU	41	20.2
Did not use PIFU	162	79.8
Total	203	100

Questionnaire results

Regarding the leaflet distribution, 183 (90.15%) patients reported receiving the informational leaflet. Among these, all patients found the leaflet easy to understand, and they felt that the instructions on how to initiate follow-up appointments were clearly explained.

Service Utilization

A total of 41 (20.2%) patients used the PIFU service; all reported being satisfied with the service. This group primarily contacted the clinic via telephone, and all the callers stated that they received appropriate and satisfactory advice, or were scheduled for an in-person appointment when necessary. Among those who did not use the service, the majority indicated they simply did not feel the need for further follow-up.

Satisfaction Ratings

When asked to rate the PIFU service, all 203 (100%) patients reported satisfaction. A total of 163 (80.3%) patients gave it a perfect rating of 5 out of 5, indicating high satisfaction. Another 20 (9.85%) patients rated the service a 4, and the remaining 20 patients (9.85%) rated it a 3. No ratings fell below a 3, reflecting an overall positive response. Patients highlighted the ease of use, autonomy, and timely responses as the most valued aspects of the service.

## Discussion

The findings from this evaluation of the PIFU service at the Royal Shrewsbury Hospital’s fracture clinic align with growing evidence supporting PIFU as a model for optimizing outpatient follow-up. The primary objective of achieving an 80% satisfaction rate was met. PIFU models allow patients to initiate follow-up appointments only when needed, an approach that reduces unnecessary follow-ups and decreases DNA occurrence, optimizing resource allocation and minimizing strain on healthcare providers [9]. PIFU is one approach to personalized follow-up care, adaptable across various specialities for patients with both acute and chronic conditions [9]. Due to the early successes, PIFU models have been suggested for implementation across various other pathways, including patients on elective surgery waiting lists [10]. In line with the NHS Long-Term Plan, PIFU has become a valuable model for managing outpatient services, especially during COVID-19 recovery, with clinicians increasingly integrating telemedicine and remote monitoring to support follow-up care [10].

In this evaluation, only 41 (20.2%) of patients assigned to the PIFU service actively engaged with it, suggesting that most felt confident self-managing their recovery. Studies have shown that PIFU can empower patients with stable conditions to self-manage effectively. For example, in a study by Hewlett et al. with patients with rheumatoid arthritis, those on a PIFU model reported lower pain levels, higher self-management confidence, and reduced healthcare costs compared to those on traditional follow-up schedules [11]. This confidence in non-engaging patients may reflect a similar comfort level with self-management, although further investigation could clarify any hesitations patients may have about initiating follow-ups independently.

Despite high satisfaction rates among PIFU users, the relatively low uptake highlights an area for potential improvement. Prior studies emphasize the importance of clear, accessible information to maximize PIFU engagement [9]. In Robinson et al.’s study on ulcerative colitis, patients in a PIFU model reported higher rates of early relapse detection and a strong preference for on-demand follow-up over traditional care [12]. Although patients in this study found the informational leaflet clear and easy to understand, providing additional education, such as through digital reminders or follow-up calls, may further bolster patient confidence in initiating contact when symptoms arise.

The positive patient satisfaction ratings in this study, with 163 (80.3%) awarding the PIFU service a top score, mirror findings from previous studies that highlight PIFU’s role in enhancing patient confidence and satisfaction. Kennedy et al., for instance, reported improved quality of life and higher satisfaction among patients managed through PIFU compared to traditional care, with fewer outpatient visits needed [13]. These findings reflect PIFU’s potential to deliver high-quality, patient-centred care while empowering patients who value greater control over their follow-up.

Moreover, by reducing routine visits and prioritizing urgent cases, the PIFU model alleviates outpatient clinic strain, making it especially beneficial in specialities such as trauma and orthopaedics where chronic conditions often require intermittent rather than fixed follow-ups [9]. This aligns with Hewlett et al.'s findings that PIFU can reduce resource costs while enhancing patient care by prioritizing accessibility for those who genuinely need follow-ups [11]. In their study on the impact of PIFU on cancer follow-up pathways, Coleridge and Morrison demonstrated a median cost-saving of £988.60 per patient, while patients individually saved an average of £57.22 on transportation and parking expenses [14]. Though complex, many of these benefits are believed to stem from enhanced patient self-management, an often under-utilized resource in healthcare. With these efficiencies, PIFU has shown promise in reducing wait times and DNAs, contributing to a more responsive clinic environment [10].

While the study demonstrates PIFU’s potential to meet outpatient care demands more flexibly, challenges remain, particularly in encouraging patients to engage confidently with the service. As seen in other settings, patients’ ability to self-manage symptoms is essential to PIFU’s success. Robinson et al., in their study on ulcerative colitis, noted that early intervention through PIFU reduced hospital visits and led to better outcomes for patients experiencing relapse [12]. Tailored education programs and additional support mechanisms, such as check-in calls, may help encourage eligible patients to engage with the service when appropriate, enhancing PIFU’s efficacy across diverse patient populations.

The limitations of this study highlight challenges in PIFU implementation and suggest caution in its broader application. Despite its potential benefits, PIFU remains rare in secondary care due to implementation barriers, the need for pathway redesign, and the shifting of responsibilities between patients and clinicians [9]. The current evidence base, while expanding, is still constrained by selection bias, small sample sizes, and limited data on long-term effects, making it difficult to fully evaluate PIFU's broader impact, particularly on primary care services, which may experience increased demands as patients self-manage more frequently in place of outpatient follow-ups. Additionally, there are concerns about losing patients who may struggle or feel hesitant to initiate follow-up; automated reminders at key intervals could help mitigate this, ensuring that patients not seen within specified windows remain engaged with care.

Another limitation involves the potential impact on vulnerable populations, such as older adults or those with limited access to digital tools, who may face greater risks of adverse outcomes under a PIFU model. Implementing additional safeguards, like shared appointment booking with a nurse or carer and enhanced education, could address these needs. Furthermore, empowering patients to recognize health changes and seek timely support is crucial; resources such as written materials, apps, and remote monitoring may improve self-management, especially for patients with multiple health conditions. However, patients with complex conditions often report confusion regarding the responsibility division between themselves and clinicians, underscoring the need for a clear delineation of responsibilities and added community support for these groups [15].

## Conclusions

In conclusion, this study demonstrates that the PIFU model can effectively reduce unnecessary outpatient appointments, alleviate clinic strain, and enhance patient satisfaction through timely, patient-centred care. With a satisfaction rate of 163 patients (80.3%) among users and active engagement from only 41 (20.2%) of eligible patients, the results highlight the PIFU model's potential to empower patients in self-managing stable conditions, provided they receive adequate guidance. Although low uptake indicates that some patients may need additional encouragement or education to utilize PIFU, satisfaction ratings and the reduced burden on clinic resources underscore its value. This evaluation suggests that refining educational materials and implementing support mechanisms, like digital reminders, may improve engagement, ensuring PIFU can be a viable and responsive alternative to traditional follow-up models in outpatient care.
